# [Retracted] MicroRNA-34a suppresses breast cancer cell proliferation and invasion by targeting Notch1

**DOI:** 10.3892/etm.2025.13055

**Published:** 2025-12-22

**Authors:** Xiaoping Rui, Haibin Zhao, Xianqiu Xiao, Li Wang, Lin Mo, Yao Yao

Exp Ther Med 16:4387-4392, 2018; DOI: 10.3892/etm.2018.6744

Following the publication of this paper, it was drawn to the Editor’s attention by a concerned reader that certain of the flow cytometric data featured in Fig. 4C on p. 4391 were strikingly similar to data appearing in different form in other articles written by different authors at different research institutes, one of which had already been submitted for publication prior to the submission of this paper to *Experimental and Therapeutic Medicine*. Moreover, concerning the cell invasion assay data shown in Fig. 5, certain of the data were also strikingly similar to data that subsequently appeared elsewhere in an article written by different authors at different research institutes, in addition to the identification of a pair of overlapping data panels within the figure itself, and the cell cycle data plots in Fig. 4A, which were intended to show the results of differently performed experiments, appeared to be remarkably similar.

In view of the fact that the abovementioned flow cytometric data had already apparently been submitted for publication elsewhere before this paper was received at *Experimental and Therapeutic Medicine*, in addition to the other problems identified with various of the figures, the Editor has decided that this paper should be retracted from the Journal. The authors were asked for an explanation to account for these concerns, but the Editorial Office did not receive a reply. The Editor apologizes to the readership for any inconvenience caused.

